# Recurrence Rate of Giant Cell Tumor With the Treatment of Scooping Curettage, Burr Down Technique, Phenolization, and Bone Cement

**DOI:** 10.7759/cureus.11953

**Published:** 2020-12-07

**Authors:** Syed Faraz Ul Hassan Shah Gillani, Yasir Iqbal, Muhammad Taqi, Tauseef Ahmad Blouch, Muhammad Iqbal, Abubakar Siddiq

**Affiliations:** 1 Orthopedic Surgery, King Edward Medical University/Mayo Hospital, Lahore, PAK; 2 Orthopedics, Swat Medical Complex and Teaching Hospital, Swat, PAK; 3 Orthopedics and Traumatology, Mayo Hospital, Lahore, PAK; 4 Orthopedics and Traumatology, Swat Medical Complex and Teaching Hospital, Swat, PAK; 5 Orthopedics and Traumatology, Lahore General Hospital, Lahore, PAK

**Keywords:** scooping curettage, burr down, phenolization, bone cementing, giant cell curettage, gct recurrence, gct treatment

## Abstract

Objective: To find the recurrence and outcomes of giant cell tumors treated with scooping curettage, burr down technique, phenolization, and bone cement.

Method: We conducted a descriptive case series using a non-probability consecutive sampling technique at the Department of Orthopedics, Lahore General Hospital, Lahore, Pakistan, from May 2014 to June 2018. A total of 40 patients aged between 20 to 40 years with Compannacci grade I, II & III giant cell tumors (GCT) were included and patients unfit for the surgery, those with multiple, recurrent, malignant giant cell tumors, tumors involving the axial skeleton, and previously treated cases were excluded. We recorded the side, site of the tumor, post-operative distal neurovascular status, and recurrence of giant cell tumors. The patients were follow-up in the out-patient department (OPD) at the second week, fourth week, 12th week, 24th week, 48th week, 96th week, and 144th week after the surgery. Side, site of the tumor, and post-operative distal neurovascular status were assessed clinically, and recurrence of the tumors was observed clinically and radiologically.

Results: The mean age of all patients was 25.75±5.74 years. Males were 45% (18) and females were 55% (22). Most (12, 30%) tumors were present in the upper limb, and 22 (70%) were present in the lower limb. The majority (24, 60%) tumors were present around the knee joint. Companacci grade I was five (12.5%), grade II was 14 (35%), and grade III was 21 (52%). There were six (15%) pathological fractures. There was no case of distal neurovascular (DNV) injury, and three patients had a recurrence in two years of follow-up.

Conclusion: Giant cell tumor treated with scooping curettage, burr down technique, phenolization and poly-methyl methacrylate showed 7.5% recurrence. The combined use of local adjuvants in the treatment of giant cell tumors is a safe and effective way to reduce the rate of local recurrence.

## Introduction

Giant cell tumor (GCT) is a locally aggressive benign tumor of the bone. It affects the young adult population with a peak incidence occurring in the third decade of life and 70% of tumors reported between 20 and 40 years. It causes joint deformity due to bone destruction and may result in disability [1]. It represents approximately 20% of all benign bone tumors [2,3]. Females have a high incidence of GCTs. Approximately 60% of GCTs are localized around the knee joint. Due to their divergent biological behavior, they are generally locally aggressive, but few can metastasize to the lung or may transform into osteosarcoma or osteofibroma [4,5].

It has characteristic multinucleated giant cells with mononuclear stromal cells. Anatomically, they are located in the diaphyseal region of the bone and present as pain, swelling, joint effusion in the affected area, and disability [6]. Surgical treatment is used for most GCT tumors [7]. Literature has reported different treatment options; including curettage that has different options such as bone grafting, cement, and bone substitution. Phenolization with various options such as bone grafting and cement. Other options include cryosurgery, radiation therapy, and embolization of the feeding vessel [8].

Data also reported intralesional curettage with high-speed burring of the cavity. This technique has been associated with a low re-occurrence rate of 12% to 25%. The addition of bone cement and phenol packing with curettage provides thermal effects and thorough removal of soft tissue [9,10]. The re-occurrence rate is 10% to 20% with high-speed burr and adjuvant therapy and 50% to 60% with conventional curettage technique [10].

The re-occurrence of GCT is a problem for treating surgeons and patients. The superiority of one treatment over the other is not clear in the literature and our population. This study aimed to determine the rate of re-occurrence of GCT tumors with scooping curettage, burr down technique, phenolization, and bone cement.

## Materials and methods

This was a descriptive case study performed using a non-probability consecutive sampling technique in the Department of Orthopedic Surgery, Lahore General Hospital, Lahore, from May 2014 to June 2018. We included 40 patients aged between 20 and 40 years of either sex with giant cell tumor of the bone diagnosed on history, clinical examination, radiography, magnetic resonance imaging, and later confirmed on biopsy. Demographic evaluation is shown in Table 1. We excluded patients unfit for surgery, recurrent, malignant giant cell tumor, or tumor involving the axial skeleton.

**Table 1:** Demographic data of the patients

variables	Frequency (N=20)	Percent (%)	
Gender of the patients Male	18	45%	
	22	55%	
Females			
Mean ages of the patients in years	25.75±5.74 years		
Limb involved Upper limb	12	30%	
Lower limb	22	70%	
Site and side of the Tumor Right Proximal Tibia	06	15%	
Right Distal Femur	04	10%	
Left Distal Femur	06	15%	
Right Distal Tibia	04	10%	
Left Proximal Tibia	08	20%	
Right Distal Radius	08	20%	
Left Distal Radius	04	10%	
Compannaci grading Garde I	05	12.5%	
Grade II	14	35.0%	
Grade III	21	52.5%	
Pathological Fracture Yes	06	15%	
No	34	85%	
Tumor extension T1	25	62.5%	
T2	15	37.5%	
Distal neurovascular injury Yes	00	00%	
No	40	100%	
Re-occurrence of tumor Yes	03	7.5%	
No	37	92.5%	

We obtained ethical approval from the institutional review board (126-13/065). Informed written consent was taken. We recorded patients’ demographic data. All patients underwent scooping curettage, burr down, phenolization, and bone cement (polymethyl methacrylate [PMMA]). Patients were under general or spinal anesthesia during the operation and all had received prophylactic intravenous antibiotics prior to and for 24 hours after the procedure. A prior biopsy was obtained through a longitudinal incision to confirm the tumor type. All procedures were performed by a single surgical team to control bias in the study. The patients were moved to the ward. Follow-up was done for two years by the researcher. Patients were followed and there was no loss to follow-up present. Radiological and clinical evaluations were performed during the follow-up period to note the recurrence rate, and all the information was recorded on a specially designed proforma. Recurrence was measured as the occurrence of giant cell tumors within 24 months after surgery with scooping curettage, burr down, phenolization, and PMMA.

We evaluated the distal neurovascular status of all patients after complete recovery from anesthesia. We removed the suction drain 24 hours after surgery and the surgical wound dressing was done. Range of motion exercises was encouraged on the first postoperative day. Weight-bearing, in the case of the lower limb, was encouraged on the first postoperative day. Tumor site, size, and recurrence were reviewed. Assessment of recurrence with specific signs - swelling, pain, pathological fracture, radiographic serial changes. A biopsy was taken for confirmation of recurrence of GCT or possible malignant transformation. All patients were followed up on the first, seventh, and 15th postoperative days and then monthly for the first year and every three months during the second year, and data were collected as per Table 2.

**Table 2:** Distribution of tumor site of patients

		Frequency	Percent
Tumor site	Left distal femur	2	10%
	Right distal femur	3	15%
	Left distal radius	2	10%
	Left proximal tibia	4	20%
	Right distal radius	4	20%
	Right distal tibia	2	10%
	Right proximal tibia	3	15%
Total		20	100%

Statistical Package for Social Sciences (SPSS) version 20.0 (IBM Corp., Armonk, NY, USA) was used to analyze the collected data. Quantitative variables such as age were presented as mean and standard deviation. Qualitative variables such as sex, site of the tumor, wound examination, and recurrence were presented as frequency and percentages.

## Results

The mean age of all patients was 25.75±5.74 years. The mean ages of male and female patients were 26±5.54 and 25.44±6.30 years, respectively. The age of male patients was higher than that of female patients. Gender distribution showed that 45% of the patients were female while 55% of the patients were male. The tumor site of the patients is given in Table 2. Two patients had a GCT tumor on the left distal femur, three patients had a tumor on the right distal femur, two patients had a tumor on the left distal radius, four patients had a tumor on the left proximal tibia, four patients had a tumor on the right distal radius, two patients had a tumor on the right distal tibia, and three patients had a tumor on the right proximal tibia. Patients' follow-up was performed from first postoperative day until 24 months. There was not a single case report of distal neurovascular injury or cortical breach during the course of follow-up, none of the patients had any kind of abnormality in the wound. All patients’ wounds were normal at the last follow-up. Only three patients had a recurrence of the tumor after surgery until the last follow-up. One recurrence appeared in the distal femur, and two were present in the proximal tibia.

## Discussion

The treatment of GCT is challenging for orthopedic surgeons due to its divergent biological behavior. The aim of GCT treatment is associated with the expectation of good functional outcomes and to avoid the risk of tumor reoccurrence. Simple curettage has a better functional outcome, however, it is associated with a high incidence of reoccurrence, while the addition of adjuvant treatment has reduced the reoccurrence rate [11]. Treatment with high-speed burr and cementation offers a low reoccurrence rate, early stabilization, and early radiological diagnosis of reoccurrence [12].

The high reoccurrence rate has caused surgeons to enhance their surgical technique with the addition of adjuvants including liquid nitrogen [13], acrylic cement [14], phenol [15], hydrogen peroxide [16], and locally administered chemotherapy. Previously, radiation therapy was associated with malignant transformation, but this concept has been challenged because of the availability of modern radiotherapy treatment [17].

The agreement on the treatment of locally recurrent tumors is controversial. The literature reported different treatment options for second or third curettage based on the pathology and aggressiveness [18]. Bone cement has been accepted as an adjuvant in the treatment of GCT with curettage and bone grafting.

When curettage is compared with bone grafting (autograft or allograft) as filling materials with bone cement, it is less expensive, provides early stability, minimal risk of joint degeneration, and early detection of GCT reoccurrence [8,10]. A study conducted in Singapore reported no reoccurrence in cases of GCT tumors treated with curettage, high-speed burring, bone cement, or adjuvant treatment. They reported a 44% reoccurrence among patients in whom adjuvant treatment was not advocated [19]. However, one study reported a 20-56% reoccurrence rate among patients who received adjuvant therapy, liquid therapy, and phenol. They reported reoccurrence in the first 30 months after the first treatment [20].

A multicenter study conducted by Turcotte et al. on 186 patients, found no significant difference in the recurrence rates when high-speed burr was compared with other local adjuvants [21].

An Iranian study in their case series of 168 patients reported 18.2% and 37.5% recurrence with curettage, high-speed burring, and curettage, respectively. According to this study, high-speed burring is an effective method for reducing recurrence [22].

Pritsch et al. treated 60 cases of GCT with extended curettage and cryosurgery. He reported a 17% fracture in his cases [23]. They never reported internal fixation in fracture cases. We performed internal fixation in our study on weight-bearing bone presenting with a fracture. All but one of our cases with the involvement of long bones in the lower extremity received plate or screw augmentation. As a result, reconstruction failed in only one patient with extensive proximal tibial involvement.

We observed 7.5% recurrence in our study among patients treated with scooping curettage, high-speed burring, phenolization, and bone cement. Our results are similar to those reported in the literature.

Our study had some limitations: small sample size, single-center, and short follow-up. Future studies may extend the findings of our study.

## Conclusions

Giant cell tumor treated with curettage, high-speed burring with the addition of adjuvant treatment with phenol and bone cement was a useful and safe method of treatment with zero recurrences, immediate stabilization, and early weight-bearing. It was also proved that the combined use of local adjuvants in the treatment of giant cell tumors is a safe and effective way to reduce the rate of local recurrence.
